# Dihydromyricetin and Salvianolic acid B inhibit alpha-synuclein aggregation and enhance chaperone-mediated autophagy

**DOI:** 10.1186/s40035-019-0159-7

**Published:** 2019-06-15

**Authors:** Jia-Zhen Wu, Mustafa Ardah, Caroline Haikal, Alexander Svanbergsson, Meike Diepenbroek, Nishant N. Vaikath, Wen Li, Zhan-You Wang, Tiago F. Outeiro, Omar M. El-Agnaf, Jia-Yi Li

**Affiliations:** 1 Institute of Neuroscience, College of Life and Health Sciences, Northeastern University, Shenyang, Liaoning China; 2 Department of Biochemistry, College of Medicine and Health Sciences, United Arab Emirates University, PO Box 17666, Al-Ain, United Arab Emirates; 3 Neural Plasticity and Repair Unit, Wallenberg Neuroscience Center, Department of Experimental Medical Science, BMC A10, 221 84 Lund, Sweden; 4 Neurological Disorders Research Center, Qatar Biomedical Research Institute (QBRI), Hamad Bin Khalifa University (HBKU), Education City, Qatar Foundation, P.O. Box 5825, Doha, Qatar; 5 Institute of Heath Sciences, China Medical University, 110112 Shenyang, People’s Republic of China; 6 Key Laboratory of Maternal-Fetal Medicine of Liaoning Province and Key Laboratory of Obstetrics and Gynecology of Higher Education of Liaoning Province, Shengjing Hospital of China Medical University, Shenyang, China; 7 Department of Experimental Neurodegeneration, Center for Nanoscale Microscopy and Molecular Physiology of the Brain, Center for Biostructural Imaging of Neurodegeneration, University Medical Center Göttingen, 37073 Göttingen, Germany; 8 Max Planck Institute for Experimental Medicine, Göttingen, Germany

**Keywords:** chaperone-mediated autophagy, macroautophagy, alpha-synuclein, protein aggregation, Parkinson disease, lysosomal-associated membrane protein

## Abstract

**Background:**

Progressive accumulation of α-synuclein is a key step in the pathological development of Parkinson’s disease. Impaired protein degradation and increased levels of α-synuclein may trigger a pathological aggregation *in vitro* and *in vivo*. The chaperone-mediated autophagy (CMA) pathway is involved in the intracellular degradation processes of α-synuclein. Dysfunction of the CMA pathway impairs α-synuclein degradation and causes cytotoxicity.

**Results:**

In the present study, we investigated the effects on the CMA pathway and α-synuclein aggregation using bioactive ingredients (Dihydromyricetin (DHM) and Salvianolic acid B (Sal B)) extracted from natural medicinal plants. In both cell-free and cellular models of α-synuclein aggregation, after administration of DHM and Sal B, we observed significant inhibition of α-synuclein accumulation and aggregation. Cells were co-transfected with a C-terminal modified α-synuclein (SynT) and synphilin-1, and then treated with DHM (10 μM) and Sal B (50 μM) 16 hours after transfection; levels of α-synuclein aggregation decreased significantly (68% for DHM and 75% for Sal B). Concomitantly, we detected increased levels of LAMP-1 (a marker of lysosomal homeostasis) and LAMP-2A (a key marker of CMA). Immunofluorescence analyses showed increased colocalization between LAMP-1 and LAMP-2A with α-synuclein inclusions after treatment with DHM and Sal B. We also found increased levels of LAMP-1 and LAMP-2A both *in vitro* and *in vivo*, along with decreased levels of α-synuclein. Moreover, DHM and Sal B treatments exhibited anti-inflammatory activities, preventing astroglia- and microglia-mediated neuroinflammation in BAC-α-syn-GFP transgenic mice.

**Conclusions:**

Our data indicate that DHM and Sal B are effective in modulating α-synuclein accumulation and aggregate formation and augmenting activation of CMA, holding potential for the treatment of Parkinson’s disease.

**Electronic supplementary material:**

The online version of this article (10.1186/s40035-019-0159-7) contains supplementary material, which is available to authorized users.

## Background

Aberrant degradation of alpha-synuclein (α-syn) has been implicated in the pathogenesis of Parkinson’s disease (PD) which leads to accumulation of α-syn in Lewy bodies [1, 2]. α-Syn can be selectively translocated into lysosomes for degradation via chaperone-mediated autophagy (CMA), a highly regulated cellular process that mediates the degradation of cytosolic proteins in lysosomes [3–6]. The protein contains a CMA target motif and is degraded by CMA in neural cells [7, 8]. CMA is controlled by two key CMA regulators: the chaperone HSC70 and the receptor lysosomal-associated membrane protein 2A (LAMP 2A). LAMP-1 is highly structurally homologous to LAMP-2A suggesting that there may be an overlapping function of these two proteins [9, 10]. HSC70 binds to protein substrates containing a KFERQ peptide motif [8, 11]. The substrate–HSC complex interacts with LAMP-1/2A for targeting of identified protein translocation to the lysosome [12–15]. In addition, the ubiquitin-proteasome system (UPS) and macroautophagy are also involved in α-syn degradation [16–18].

Several studies have been conducted on the development of small molecular inhibitors of α-syn aggregation for the prevention and treatment of PD [19–23]. Several important compounds in the daily diet and medicinal plants have been found to be protective against α-syn fibrillation [21, 24]. Dihydromyricetin (DHM), a major active ingredient of flavonoid compounds extracted from the stems and leaves of *Ampelopsis grossedentata*, has anti-tumor, oxidation resistance and free radical scavenging capabilities [25–28]. Evidence indicates that DHM has neuroprotective effects by enhancing the formation of autophagosomes and inducing autophagy [29–31]. Salvianolic acid B (Sal B) is one of the bioactive compounds of *Salvia miltiorrhiza Bunge* extracted from the root of Salvia miltiorrhiza and has been shown to exert various anti-oxidative and anti-inflammatory effects in both *in vitro* and *in vivo* studies [23, 32, 33]. Sal B has recently been associated with preventing fibril aggregation of amyloid proteins and inhibiting neuroinflammation, thereby improving neurological function in animal models of neurodegenerative diseases [23, 24]. However, it is not clear whether DHM and Sal B have any effects on α-syn accumulation and aggregation in synucleinopathies, such as PD.

To further explore the role of CMA mediated degradation of aggregated α-syn and the potential function of autophagy regulated by DHM and Sal B, in the present study, we have investigated the effects of DHM and Sal B on α-syn accumulation and aggregation using both *in vitro* and *in vivo* models. We observed that DHM and Sal B upregulated the CMA associated protein LAMP-2A and its homologous protein, LAMP-1, decreased levels of α-syn, reduced cytotoxicity and inhibited inflammatory responses when administered in cell and animal models. Our findings indicate that DHM and Sal B are potential therapeutic compounds that can intervene and halt pathological developments in synucleinopathies.

## Methods

### Fibril preparation

α-Syn monomers were ordered from Proteos (RP-003) and prepared following the Michael J Fox Foundations guidelines for fibril formation. Briefly, monomeric protein was thawed and spun at 15.000xg for 10 min at 4 °C, to pellet any aggregated materials. The supernatant was then assessed by BCA to determine the α-syn concentration. The monomer sample was diluted to 5 mg/ml in PBS without calcium and magnesium, and transferred to a 1.5 ml Eppendorf tube, then incubated for 7 days in a shaking incubator at 1000 rpm and 37 °C. Final fibril solution was stored at -80 °C in single use aliquots until use.

### Inhibitor modulation of α-synuclein aggregation kinetics

Aggregation kinetics were assessed in Corning NBS half-area micro plates(#3881) plates using 70 μM α-syn monomers, 20 μM thioflavin T (Sigma, T3516) and Sal B (Sigma, SML0048, ≥94% (HPLC) ) or DHM (Sigma, SML0295, ≥98% (HPLC) ) at either 15 or 30 μM. Vehicle control wells were set up using a volume equal to that of the highest inhibitor concentration (0,015 μl per well). To initiate the experiment, sonicated α-syn seeds were added to each well at 0.1% of the monomer concentration (70 nM). Kinetics were observed using a BMG FLUOstar Omega plate reader, allowing continual measurements for 7 days at 37 °C. Baseline acquisition was performed for 3 hours before addition of the α-syn seeds, and recordings were continued for 12 hours.

### Cell culture and transfection

H4 neuroglioma cells from human (origin) were cultured in Opti-MEM + GlutaMAX (Invitrogen, 51985-034) supplemented with 10% fetal bovine serum (FBS; Gibco, 10100-147) at 37°C, passaged, and plated on chamber slides (Labted-II, Nalgen-Nunc, 154526) or glass cover slips. For intracellular α-syn aggregation experiments, H4 cells were seeded in 24-well plate (5 × 10^4^ cells/well) 24 h prior to transient transfection with SynT (C-terminal tagged form of WT α-syn) and synphilin-1 (Fig. S1). Equi-molar ratios of plasmids were mixed with FuGENE® 6 (Promega, E2691) at a 1:2 mass volume ratio, and incubated for 15 min before the complex of transfection reagent and plasmids was transfected into cells according to the manufacturer’s protocol (2 h transfection and 6 h recovery time). ALP modifiers were incubated during the last 24 h before fixation and processing for immunocytochemistry and toxicity assessments. Co-transfection with an empty backbone-vector [pPAGFP-C1, Addgene, 11910] and mock transfection was used as control. Rapamycin (200 ng/ml, Sigma Aldrich, R0395) was prepared in DMSO, chloroquine diphosphate salt (50 mM, CQ, Sigma Aldrich, C6628) and 3-methyladenine (10 mM, 3-MA, Sigma Aldrich, M9281) in water.

### Immunocytochemistry

For drug treatment, 24 h post first transfection with SynT and synphilin-1, cells were further incubated with DHM, Sal B, Rapamycin or Chloroquine. Twenty-four hours later, cells were fixed with 4% PFA for 10 min at room temperature (RT), washed two times with PBS and subjected to immunocytochemistry analysis. Briefly, cells were permeabilized with 0.5% Triton X-100 in PBS for 20 min at RT, blocked for 1 h at RT with 5% normal donkey serum in 0.1% Triton X-100 in PBS, incubated with primary antibody (mouse anti-α-syn 1:1000; BD Biosciences) at 4°C overnight followed by secondary antibody incubation (1:1000, donkey anti-mouse IgG-Alexa568, Jackson ImmunoResearch) for 2 h at RT, then incubated for 10 min with DAPI 1:1000 in PBS (SIGMA-ALDRICH). Specimen analyses were performed with a conventional epifluorescence microscope (Nikon Ni-E). Cells were subjected to microscopy analysis for LAMP-1/-2A and α-syn colocalization using laser-confocal microscope (Leica TCS SP8), followed by analysis using ImageJ software. Sequential multi-track frames were acquired to avoid any potential crosstalk from adjacent fluorophore. For ThS labeling, transfected H4 cells were fixed and incubated prior to IF labeling for 10 min in 0.5 mg/ml thioflavin-S (Sigma, T1892), and washed in 85% absolute ethanol. At least 300 cells from three independent wells were assessed for each experiment and the number of cells containing α-syn positive aggregates were quantified in the transfection conditions by a random sampling survey. The percentage of the transfected cells containing α-syn positive aggregates compared with the total number was then recorded.

### Immunohistochemistry of transgenic mice

Six- and nine-month old mice were housed (3-4 animals/cage) with food and water available *ad libitum* under a 12-h light/dark cycle. All animal experiments followed the Institutional Animal Care and all procedures were performed under the specifications set by the Ethical Committee for Use of laboratory animals at Lund University, Sweden and at Northeastern University, China. Homozygous transgenic mice expressing WT human α-syn fused to green fluorescent protein (GFP), under control of the mouse α-syn promoter show an overexpression of α-syn-GFP in the CS and the dopaminergic neurons of the SNpc. The formation of α-syn aggregates in the brain of transgenic mice has been shown to rise with increasing age [34]. DHM and Sal B (10mg/kg/day for two weeks) were utilized for intra-peritoneal administration of nine-month old mice (n=8 mice per group). Mice were then euthanized 6 weeks later. Brains were removed, post-fixed in 4% PFA and a gradient sucrose sedimentation (10% - 30%) was performed. For mouse brains, 30 micrometer-thick free-floating coronal sections were cut on a freezing microtome (Leica, SM2010R), blocked in solution comprising PBS + 5% horse serum + 0.25% Triton-X 100, and incubated with primary antibodies (mouse-anti-α-syn antibody 1:1000, Santa Cruz Biotechnology, sc-12767; mouse-anti-GFAP antibody 1:1000, MERCK MILLIPORE, MAB360; mouse-anti-Iba1/AIF1 antibody 1:1000, MERCK MILLIPORE, MABN92) overnight in a humid chamber at 4°C. Sections (incubated with anti-α-syn, anti-GFAP and anti-Iba1/AIF1) were then subsequently incubated with secondary biotinylated anti-mouse antibody (Vector Biolabs) followed by DAB staining using the ABC kit (Vector Biolabs) and DAB peroxidase substrate (Vector Biolabs) according to the manufacturer’s protocol. For each animal, 3 sections were analyzed and all sections were processed under the same standardized conditions. α-Syn^+^, GFAP^+^ and Iba1^+^ cells in the CS and SNpc were counted on a Nikon microscope using the NIS-Elements BR imaging system.

### Western blot analysis

Cytosolic fractions were obtained by manual homogenization and incubation in ice-cold lysis buffer containing 25 mM TRIS-HCl pH 7.4, EDTA 1 mM, protease inhibitor + 0.1% SDS for 2 h, followed by centrifugation at 12000 rpm for 10 min. In the supernatant, equivalent amounts of LAMP-1/-2A protein sample were loaded and separated by 8% SDS-PAGE gels, and transferred to polyvinylidene difluoride (PVDF) membranes (Millipore) for 2 h at 4°C or overnight at 4°C. Membranes were blocked with 2.5% nonfat milk solution in Tris-buffered saline with 0.1% Triton X-100 (TBST) for 1 h, and then incubated overnight at 4°C with mouse anti-LAMP-1 (Abcam, ab25630, 1:1000), rabbit anti-LAMP-2A (Abcam, ab18528, 1:1000) or mouse anti-β-actin (Sigma, A1978, 1:5000,), followed by HRP-linked secondary antibodies (CST, 7076S for anti-mouse IgG and 7074S for anti-rabbit IgG, 1:10000,) for 2 h at RT. Bands were detected using an ECL detection kit (Cell Signaling Technology) and exposed to X-ray films. Bands were analyzed and normalized to the corresponding β-actin signal for comparison.

### Cytotoxicity assays

Cell viability and α-syn cytotoxicity was evaluated by the MTT assay and LDH assay. H4 cells were plated on 96-well plates in complete medium, transfected with SynT and synphilin-1, then co-transfected with a plasmid encoding for WT α-syn, or with empty plasmid. For the MTT assay, 10 μl MTT reagent and 100 μl detergent reagent were added into each well in sequence after incubation for twenty-four hours. The resulting intracellular purple formazan can be solubilized and quantified by spectrophotometric means. For the LDH assay, after twenty-four hours transfection, culture media were collected and used to determine the levels of released Lactate dehydrogenase (LDH). After treatment, assays were performed following the manufacturer’s instructions (Promega, Madison, WI, USA). Results were expressed as the percentage of cell death.

### Open Field Assay

Exploratory/locomotor activity of the animals (six-month-old and nine-month-old, n=8 mice per group) was assessed in an open-field paradigm equipped with a video trajectory analysis system (42 × 42 × 36 cm [d × w × h] plexiglass boxes) and analyzed using Smart 3.0 software (Panlab, Span). The mice were first allowed to explore the confined arena for 15 min during each session, then their performance was recorded and ambulatory locomotor activity was measured by offline analysis. Track paths were subsequently analyzed by an automatic system to assess the following parameters: distance traveled (cm), time spent in the various sections of the arena and number of rears.

### Data analysis and statistics

Statistical analyses were performed using Prism 6 (GraphPad Software). All data shown are representative from a minimum of three independent experiments unless otherwise stated. Statistical analysis for comparison of groups in the *in vitro* experiments was performed using the Student’s *t*-test. For both *in vitro* and *in vivo* experiments, statistical significance of difference between groups was determined by the 2-tailed unpaired Student *t* test of the means. Where values have been compared with the normalized control, a one-sample *t* test was used. In cases of multiple-group comparisons, a one-way ANOVA was used, with Scheffe’s *post hoc* test where values have been compared with the control.

## Results

### Aggregation kinetics of α-synuclein in the presence of Sal B and DHM

**Fig. 1:**
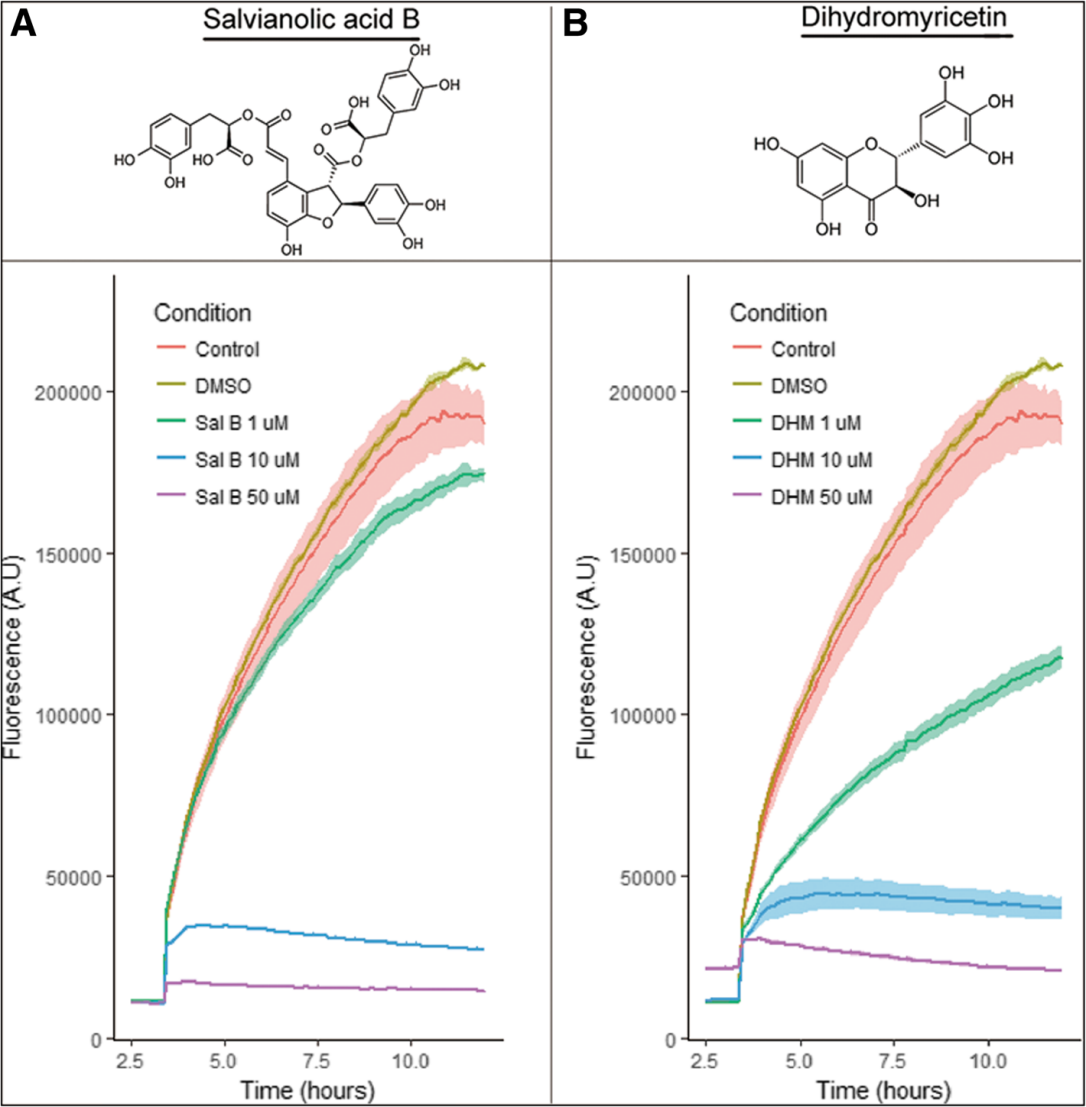
Sal B and DHM inhibit seeded aggregation of α-syn in a concentration dependent manner. Chemical structures of Sal B (**a**) and DHM (**b**) as used throughout the study. Seeded α-syn aggregation can be inhibited by addition of either Sal B (**a**) or DHM (**b**) in increasing concentrations. A baseline of ThT fluorescence was captured for both A and B for 3 hours. For both Sal B and DHM, a concentration of 1 μM slows the aggregation kinetics, while higher concentrations lead to a complete inhibition of aggregation process. Graphs are plotted as median of 3 replicates (±95% Confidence Interval)

We first studied the aggregation kinetics of α-syn in the presence of Sal B and DHM in increasing concentrations using the thioflavin T (ThT) fluorescence assay. ThT can bind to the β-sheet structure of α-syn and changes in the fluorescence signal of this dye are used to monitor the formation of α-syn fibrils. The inhibiting effects of Sal B or DHM on the aggregation kinetics of α-syn were monitored by starting the fibrillation process in the absence and presence of Sal B or DHM while continually measuring the ThT fluorescence from the fibrillation process using the plate-based assays with ThT dye (Fig. 1). Following addition of pre-formed fibrils (PFF) of α-syn, an increase in the ThT fluorescence indicates α-syn conversion from monomer to β-sheet rich aggregates (Fig. 1a, b). The results suggest that Sal B and DHM can effectively inhibit α-syn aggregation in a concentration-dependent manner (10μM and 50 μM for both Sal B and DHM).

### Alterations of CMA markers in an *in vitro* model of α-synuclein aggregation.

In order to study alterations in α-syn aggregation *in vitro*, we performed a co-transfection of SynT and synphilin-1 in human neuroglioma cells (H4 cells) for at least 48 h, leading to α-syn aggregation [35] (Additional file 1). As we reported previously, in this aggregation model we detected many large α-syn aggregates in contrast to wild-type (WT) α-syn transfected cells, where diffuse α-syn was present in the cytoplasm (Additional file 1Bi, Ci). In addition, the cells transfected with SynT exhibited large ThS positive aggregates, implying that α-syn aggregates consist of a typical β-sheet conformation in the cells (Additional file 1Bii). Small and diffused α-syn positive structures were also found in each model but were not ThS positive (Additional file 1Cii), in agreement with our previous findings [36].

**Fig. 2:**
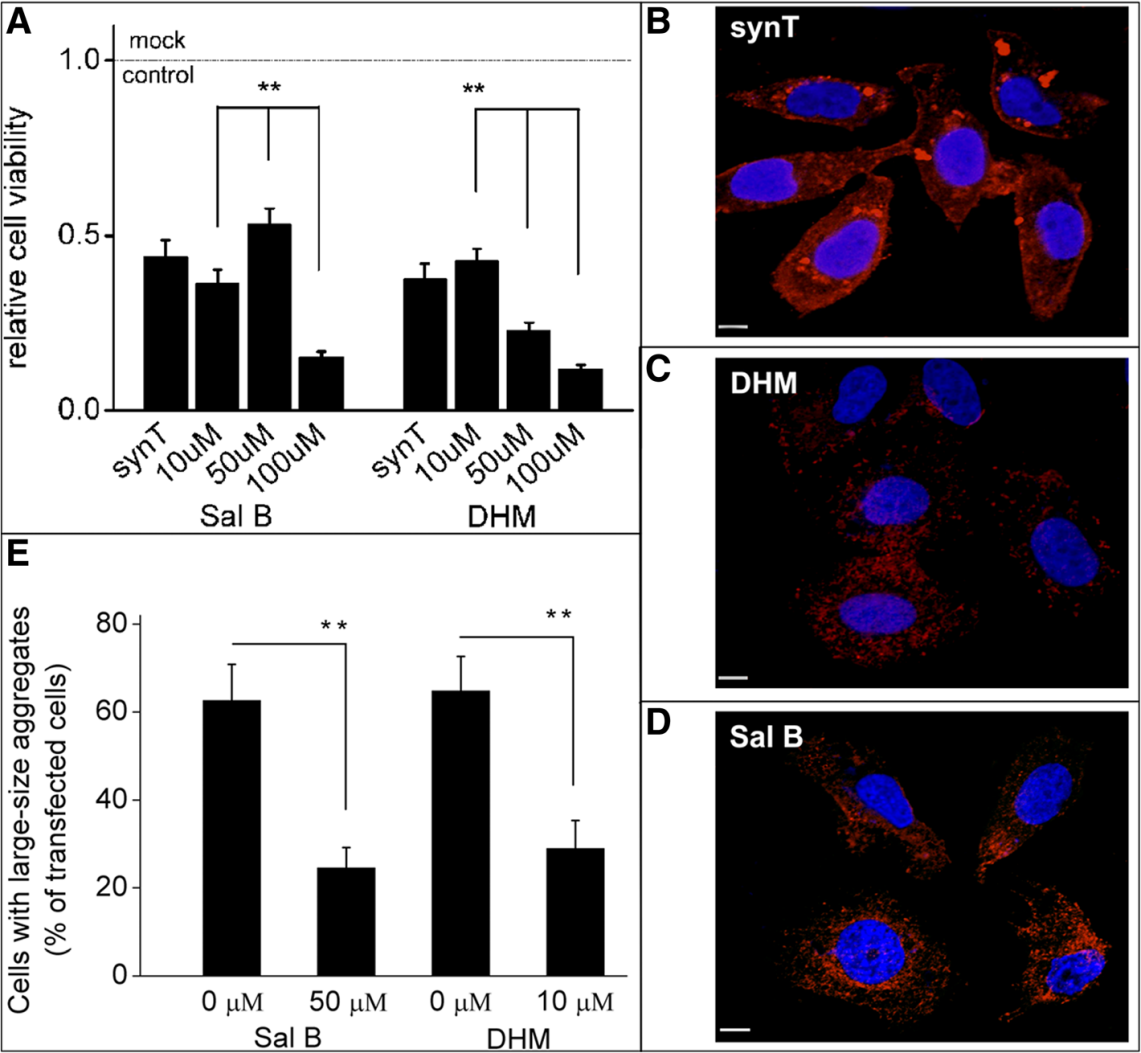
The effects of small molecule drugs on α-syn aggregation in H4 cells. (**a**) MTT assay was performed in a dose-dependent manner in SynT and synphilin 1 co-transfected H4 cells. IF labeling for α-syn (red) showed a greater quantity of large-sized aggregates in untreated cells (**b**) than that in DHM or Sal B treated cells (**c**, **d**, **e**). All data shown are representatives of at least three independent experiments (mean ± SD, *p<0.05, **p<0.01), Scale bar = 5 μm

**Fig. 3:**
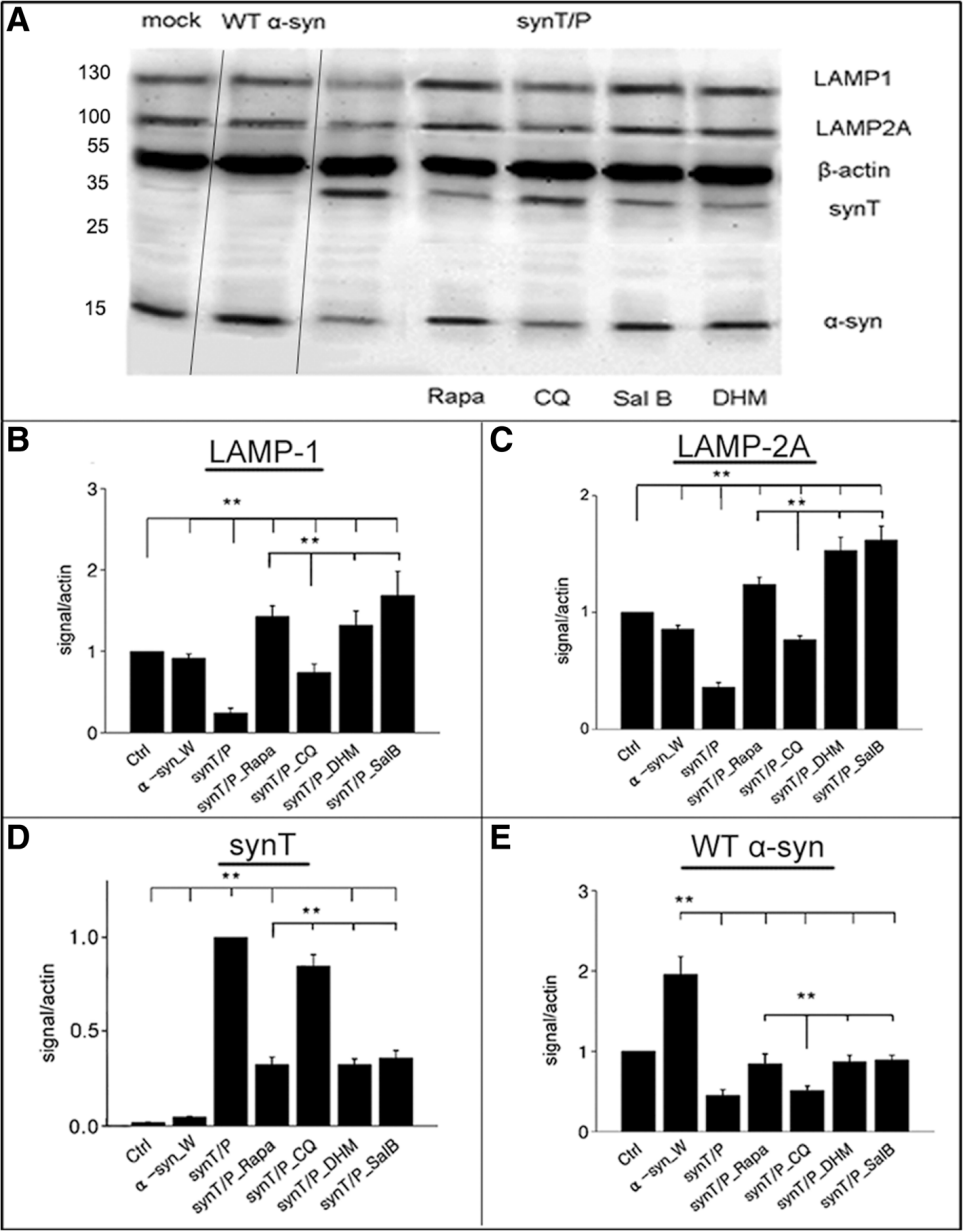
Expression of LAMP-1 and LAMP-2A in α-syn transfected H4 cells. A control group without any transfection was performed as a ‘mock’ while another group with an ordinary wildtype α-syn plasmid transfection was performed as ‘WT α-syn’. SynT and synphilin 1 co-transfected group was performed as the experimental group with DHM, Sal B, Rapamycin or Chloroquine treatments. The expression levels of LAMP-1/-2A, SynT or endogenous α-syn were detected by Western blot (**a**). Quantitative analyses of α-syn (SynT) and LAMP-1/-2A normalized to β-actin (B-E) (n = 5). Expression of LAMP-1 (**b**) and LAMP-2A (**c**) was significantly increased after treatment with DHM or Sal B, as compared with WT α-syn transfected cells, rapamycin treated cells or CQ treated cells (after normalization to untreated cells). (**d**) SynT levels were decreased in both DHM and Sal B treated cells as compared to the SynT-aggregation model or (CQ) treated cells. (**e**) WT α-syn expression levels were increased in DHM (10 μM) or Sal B (50 μM) treated cells as compared with the SynT-aggregation model. All data shown are representative of at least three independent experiments (mean ± SD, *p<0.05, **p<0.01)

To further study the effects of DHM and Sal B on α-syn aggregate-degradation in the cells, we used an H4 cell model expressing SynT-aggregation. First, we performed an MTT assay in a dose-dependent manner to optimize the doses of DHM and Sal B. The optimal doses were selected as 10 μM and 50 μM for DHM and Sal B, respectively (Fig. 2a) and the optimal treatment time for both DHM and Sal B was 16 h (data not shown). Morphological analysis of immuno-labeled α-syn-positive structures revealed that larger inclusions (2-5 μm in diameter or larger, Fig.2b) were found in untreated cells than that in DHM or Sal B treated cells (1 μm in mean diameter, Fig. 2c, d and e). Quantitative analysis of CMA marker expression in the SynT-aggregation model by SDS-PAGE and Western blotting revealed that LAMP-1 and LAMP-2A were significantly increased in DHM and Sal B treated cells compared to the cells with empty vector, SynT and WT α-syn transfection (Fig. 3a, b and c), while the expression levels of α-syn were significantly reduced by treatment with DHM and Sal B (Fig. 3a, d). No significant differences for WT α-syn levels were found in any of the cell models except for the SynT-aggregation model (Fig. 3a, d and e). Rapamycin can inhibit the mammalian target of rapamycin (mTOR) pathway and can activate the autophagy-lysosome pathway (ALP) [37]. Chloroquine (CQ) can reduce the fusion of autophagosomes with lysosomes and inhibit the ALP [38]. Therefore, we treated cells expressing SynT with either rapamycin or CQ as positive and negative controls, respectively. We found that the expression pattern of WT α-syn, SynT, LAMP-1 and LAMP-2A were similar in rapamycin treated cells and in DHM and Sal B treated cells. In CQ treated cells, the levels of WT α-syn, LAMP-1 and LAMP-2A were significantly reduced and the levels of SynT were significantly increased as compared to the DHM and Sal B treated cells (p<0.05, Fig. 3). In order to test the involvement of DHM and Sal B in macroautophagy, we treated the SynT-transfected cells with 3-MA as an inhibitor of macroautophagy. The treatment with 3-MA alone resulted in a reduction of LC3-II levels, whereas an increase in expression levels of LC3-II and LAMP-1/2A was observed after DHM or Sal B treatment (Additional file 2). These findings suggest that DHM and Sal B lead to an up-regulation of the LC3-II and LAMP-2A proteins reflecting the involvement of CMA and macroautophagy after treatments with DHM and Sal B.

**Fig. 4:**
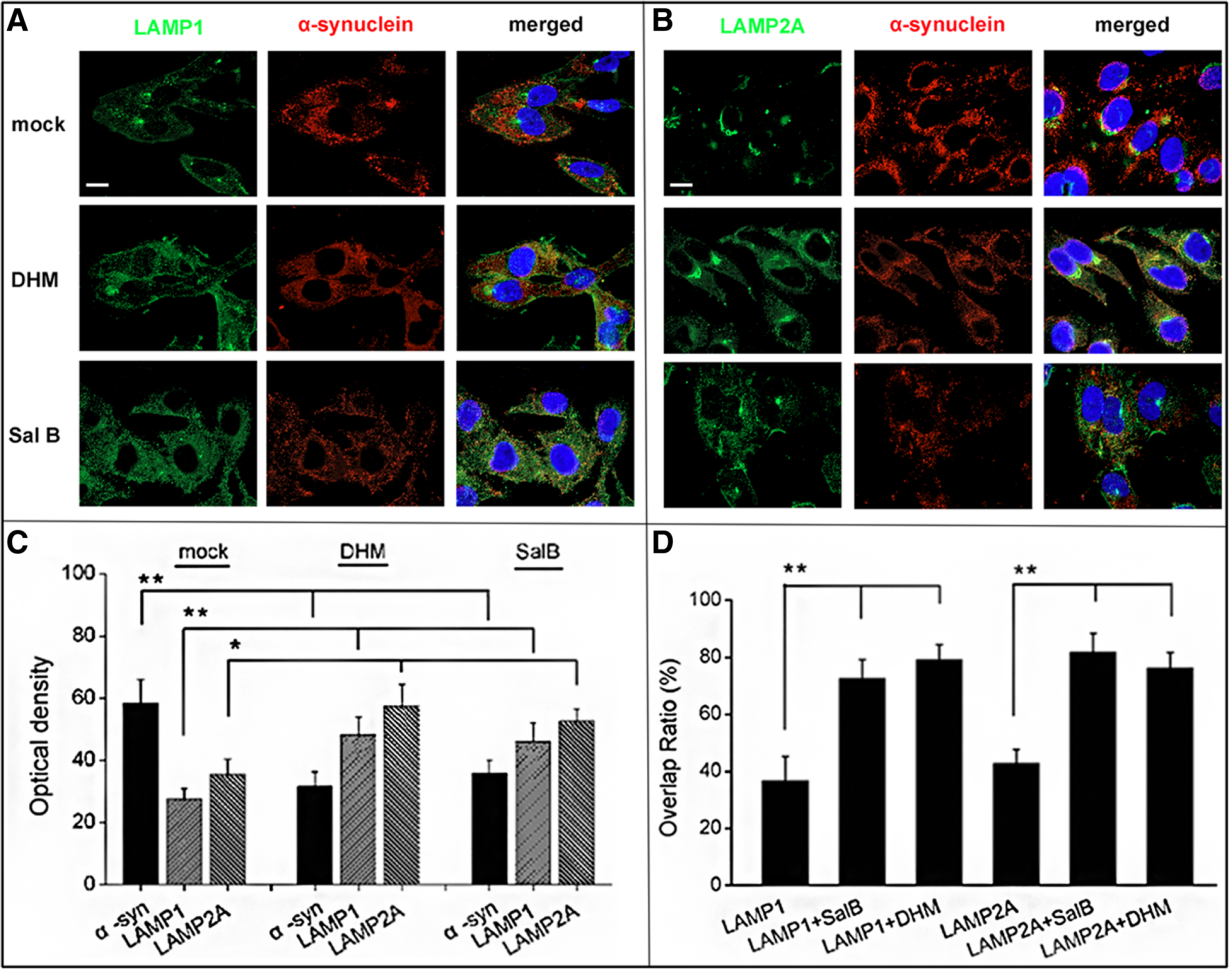
Drug effects on α-syn aggregates and co-localization of LAMP-1, LAMP-2A in the SynT-aggregation model. H4 cells were co-transfected with SynT and synphilin-1. (**a**-**b**) Cells were fixed 48 h post-transfection and subjected to IF for α-syn (red) and LAMP-1/-2A (green) followed by confocal microscopic analysis. (**c**) LAMP-1 and LAMP-2A showed an increased expression level in SynT transfected H4 cells following treatment with DHM (10 μM, 48h) and Sal B (50 μM, 48h), compared to untreated cells. The fluorescence density levels of α-syn were decreased in DHM and Sal B treated cells compared with the control. (**d**) The co-localization levels of LAMP-1/-2A with α-syn in intracellular aggregates were increased in DHM and Sal B treated cells. All data shown are representative of at least three independent experiments (mean ± SD, *p<0.05, **p<0.01), Scale bar = 5 μm

Next, we examined the effects of DHM and Sal B on α-syn aggregation and the interaction with LAMP-1 and LAMP-2A. Interestingly, we observed that α-syn inclusions co-localized with LAMP-1 and LAMP-2A in more defined regions in cells treated with DHM and Sal B (Fig. 4a, b and d), whereas the untreated cells showed much less co-localization between α-syn and LAMP-1 or LAMP-2A (Fig. 4d), suggesting a potential relationship between the α-syn inclusions and the CMA pathway. Quantification of the signals of α-syn and LAMP-1/-2A revealed that DHM and Sal B treatments increased the levels of LAMP-1/-2A and mitigated α-syn aggregation. Quantification of the mean fluorescence intensity revealed a significant increase of around 60%-70% for LAMP-1 (+75% ± 10.5% SEM for DHM treated group; +58% ± 9.8% SEM for Sal B treated group) and LAMP-2A (+63% ± 10.5% SEM for DHM treated group; +52% ± 8.7% SEM for Sal B treated group) levels in the lysates of SynT-aggregation H4 cells compared to the untreated group (Fig. 4c). The expression level of α-syn was reduced in the DHM and Sal B treated cells (by 77% ± 14.1% SEM for DHM treated group; 68% ± 11.8% SEM for Sal B treated group) compared to the untreated group. Thus, the altered levels of the lysosomal membranes (LAMP-2A) support the association between α-syn aggregation and CMA pathway activation, particularly in response to DHM and Sal B administrations.

### CMA modulates α-synuclein aggregation and toxicity *in vitro*

**Fig. 5:**
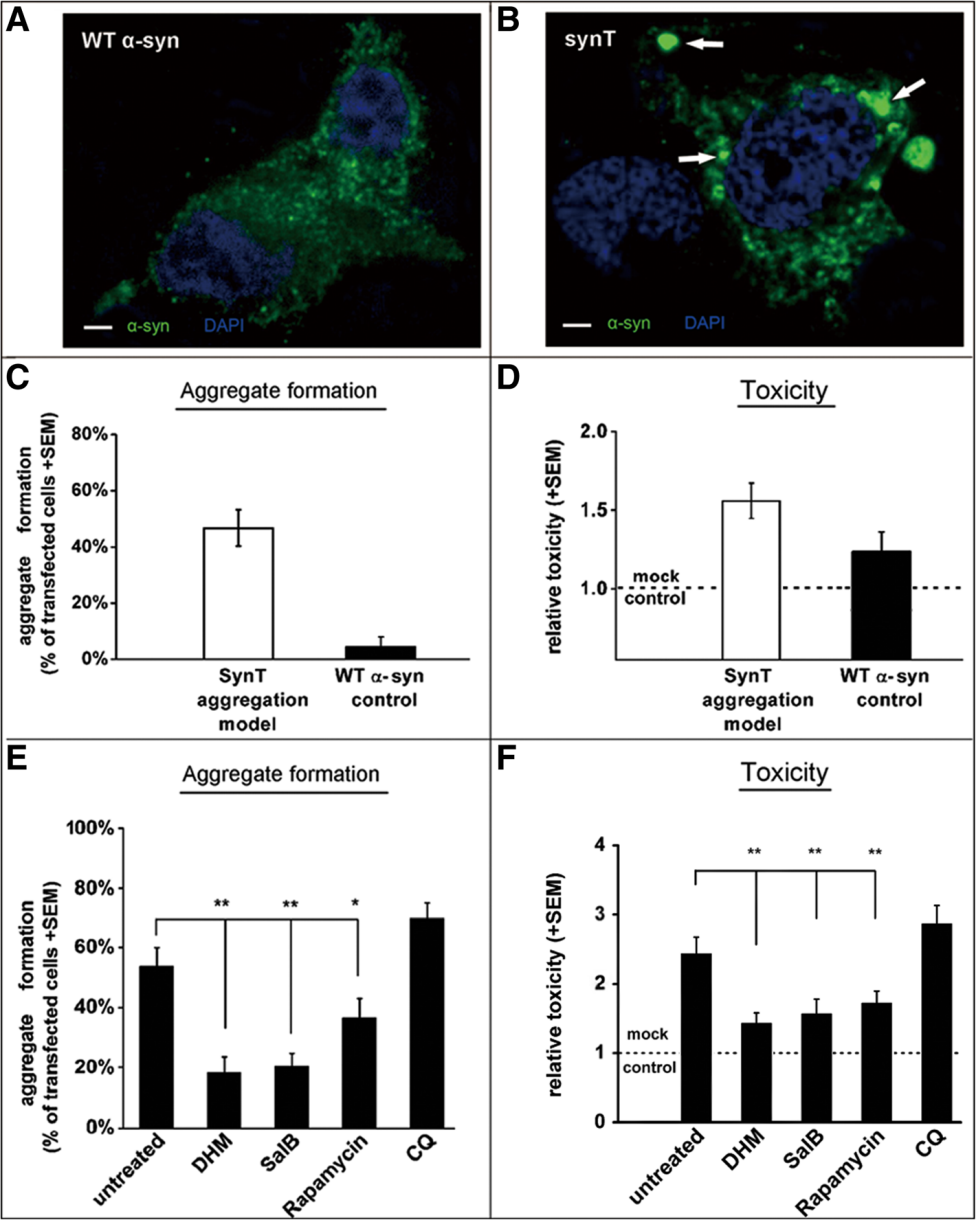
Molecules modulate the cytotoxicity of α-syn aggregation in cells transfected with WT α-syn or co-transfected with SynT and synphilin-1. Transfection with WT α-syn did not induce clear aggregate formation (green, **a**), while transfection with SynT leads to distinct α-syn positive inclusions (arrows, B) in 50–60% of transfected cells (**c**). Nuclei are stained in blue (**a** and **b**). (**d**) Toxicity, as measured by the release of Lactate Dehydrogenase in transfected cells, showed no significant difference between the SynT-aggregation model (*n* = 6) or WT α-syn control (*n* = 5). Treatment with DHM or Sal B in SynT transfected cells reduced the number of cells with SynT-inclusions (**e**, **p<0.01), and ALP activation with rapamycin also showed a decrease in α-syn aggregation in transfected H4 cells (**e**, *p<0.05). The number of cells with SynT-aggregates was slightly increased after treatment with CQ. (**f**) Treatments with DHM, Sal B or rapamycin showed a significant decrease in cell toxicity (**f**, **p<0.01), in contrast to the SynT transfected group, or the CQ treatment group. Scale bar = 5 μm

The findings above suggest that CMA modulation may be a potential target for intervention in synucleinopathies. Thus, we investigated whether the effects of α-syn aggregation and toxicity are regulated by CMA (Fig. 5). Although α-syn inclusions were present in approximately 50% of the SynT transfected H4 cells (Fig. 5a, c), the toxicity was not significantly increased compared with that observed in cells expressing WT α-syn (Fig. 5b, d). Both DHM and Sal B treatments decreased the percentage of aggregate-containing cells (SynT transfected H4 cells) from 52.1% (± 10.1% SEM) to 18.2% (± 8.3% SEM, p<0.01, DHM) and 19.6% (± 6.7% SEM, p<0.01, Sal B), respectively, compared with rapamycin treated cells (to 33.8% ± 8.6% SEM, p<0.01) (Fig. 5e). This was paralleled by significant decreases in toxicity by 1.48-fold (± 0.28 SEM, DHM), 1.65-fold (± 0.34 SEM, Sal B) and 1.78-fold (± 0.31 SEM, rapamycin) compared to the SynT transfection model (2.47 fold (± 0.32 SEM)) (Fig. 5f). Thus, these results suggest that DHM and Sal B have ameliorative effects on α-syn aggregation and attenuate toxicity for aggregation-prone α-syn species.

### DHM and Sal B treatments activate CMA pathways and degrade α-syn aggregates *in vivo*.

**Fig. 6:**
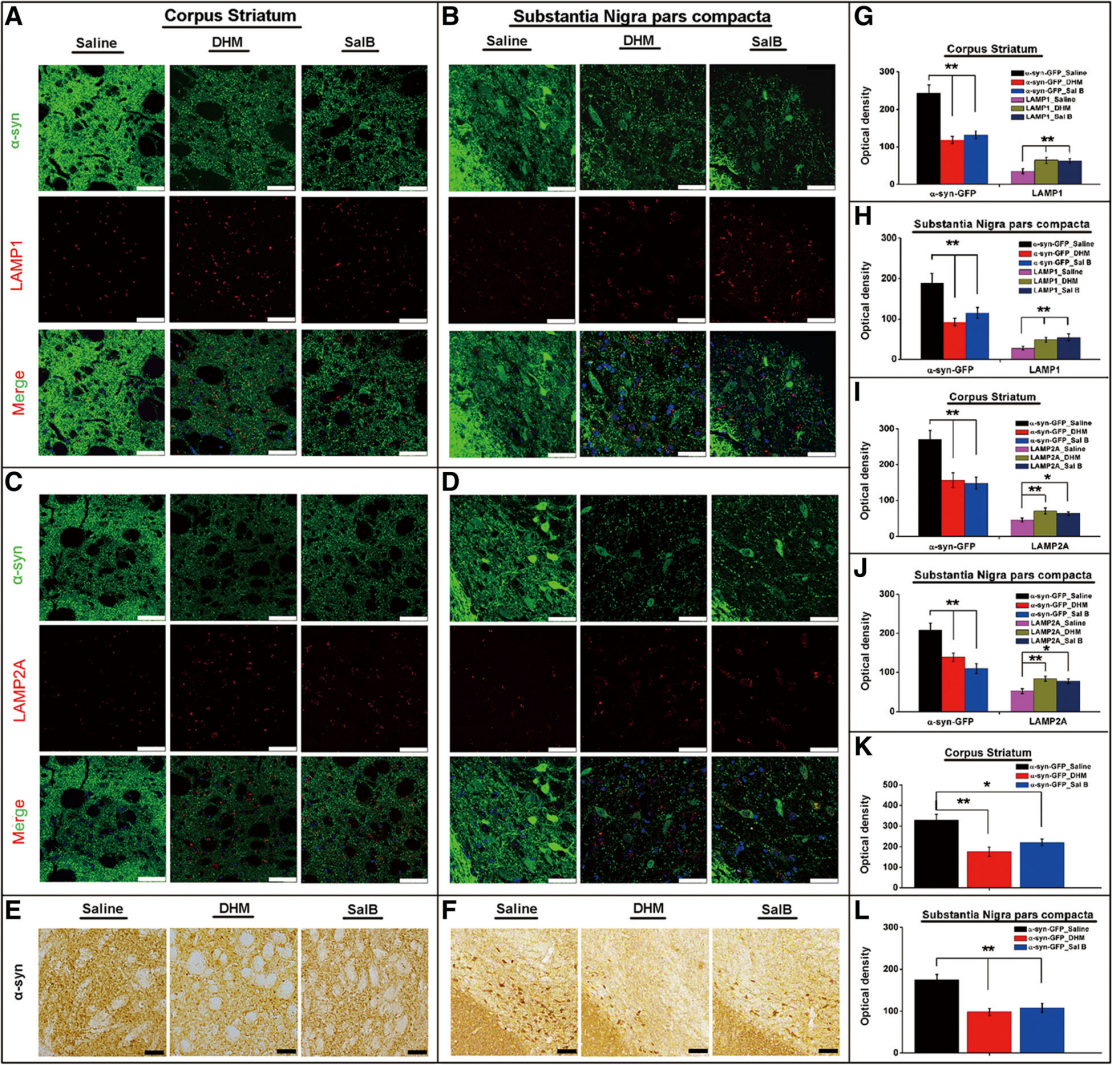
LAMP-1 and LAMP-2A expression patterns in BAC-α-syn-GFP transgenic mice upon DHM and Sal B treatments. Morphological and quantitative analyses of α-syn-GFP in the CS (**a**, **c**) and SNpc (**b**, **d**) of BAC-α-syn-GFP transgenic mice showed decreased accumulation of α-syn-GFP, but an increased presence of LAMP-1 and LAMP-2A in DHM/Sal B treated groups compared with the saline treated group (8 animals/group, male). Similar results were observed in immunohistochemcally stained CS (**e**) and SNpc (**f**) of BAC-α-syn-GFP transgenic mice treated with DHM/Sal B or with saline control. Quantitative analyses of optical density of α-syn-GFP, LAMP-1 and LAMP-2A are displayed in the right panel (**g**-**j** for IF intensity, **k** and **l** for the intensity of immunoreactivity). Significant differences are indicated (*p<0.05, **p<0.01) as compared with the saline-treated group. Scale bars = 50 μm

To validate the effects of DHM and Sal B treatments *in vivo*, we administered them to Bacterial Artificial Chromosome (BAC) transgenic mice, expressing WT human α-syn fused to green fluorescent protein (GFP) under the control of the endogenous α-syn promoter [34]. We observed a widespread expression of α-syn-GFP in multiple brain regions, including the *corpus striatum* (CS) and *substantia nigra pars compacta* (SNpc). To study the effects of DHM and Sal B on mouse behavior, we assessed locomotor activity in an open field test using 6 and 9-month-old mice. Upon administration of DHM (10 mg/kg/day), Sal B (10 mg/kg/day) or vehicle for two weeks, we did not observe any significant differences in ambulatory movements among the groups of WT vs transgenic mice (treated with DHM, Sal B or vehicle) (Additional file 3). We then examined the expression of α-syn and LAMP-1/-2A in DHM and Sal B treated groups compared to the saline treated group in the BAC-α-syn-GFP transgenic mice. Morphological analyses with IF and immunohistochemical (IHC) preparations revealed that in the CS and SNpc, the levels of α-syn-GFP (IF) (Fig. 6a-d, g-j) and α-syn (IHC) (Fig. 6e, f, k, l) significantly decreased upon administration of DHM and Sal B, while the levels of LAMP-1 (Fig. 6a, b, g, h) and LAMP-2A (Fig. 6c, d, i, j) significantly increased, compared with the saline control group (p<0.05) (Fig. 6a-f, left column), suggesting that DHM (Fig. 6a-f, middle column) and Sal B (Fig. 6A-F, right column) treatments activated CMA pathways in the brains of transgenic mice. Furthermore, we found that LAMP-1, LAMP-2A and α-syn-GFP exhibited a co-localized distribution in some subcellular compartments in the DHM and Sal B treated mice (Additional file 4), further indicating increased degradation of α-syn aggregates via the CMA pathway.

**Fig. 7:**
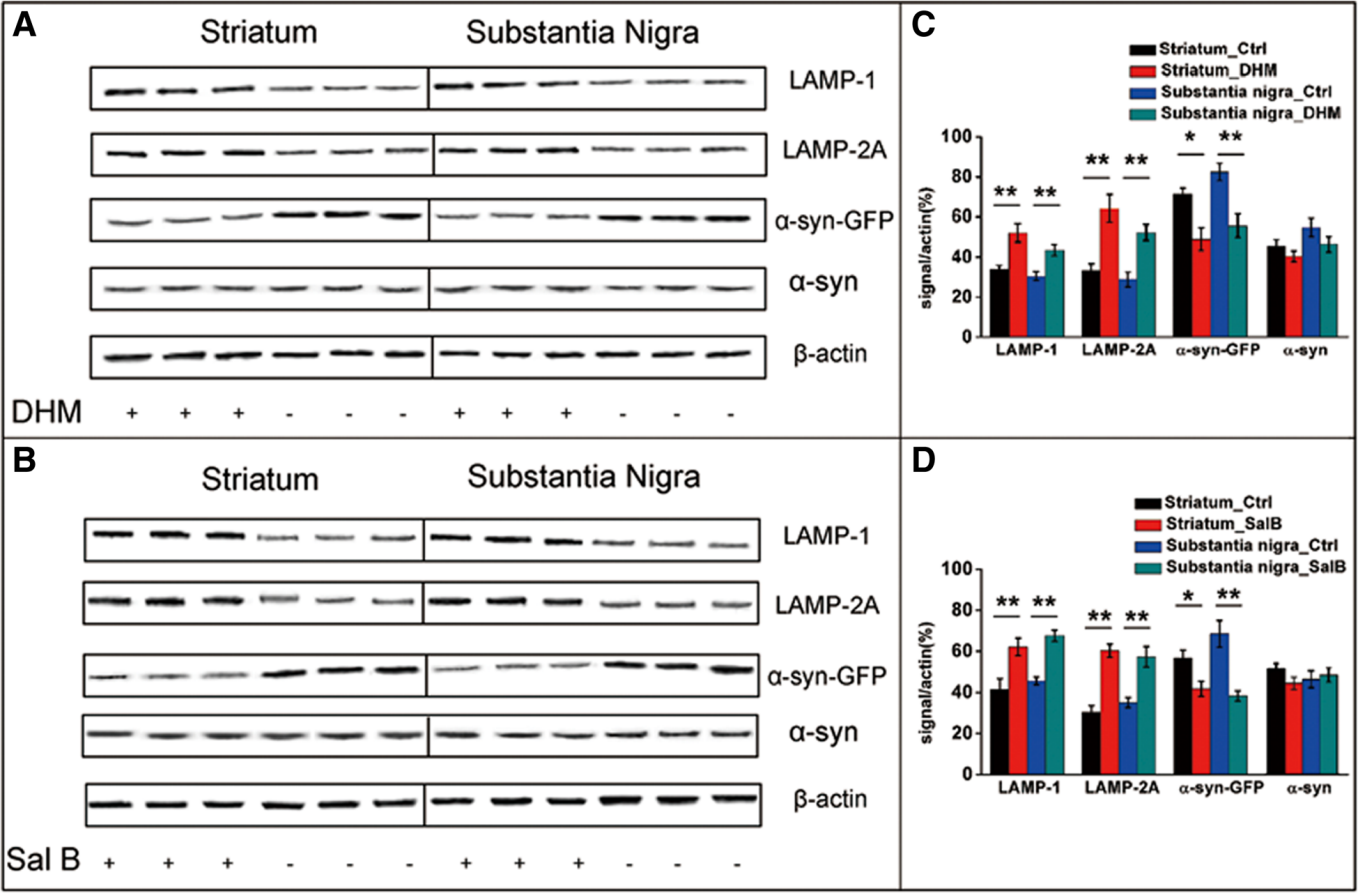
Levels of LAMP-1/-2A, α-syn-GFP and (mouse endogenous) α-syn in BAC-α-syn-GFP transgenic mice. Representative Western blots (**a**, **b**) and intensity quantification (**c**, **d**) of related proteins normalized to actin are shown (8 animals/group, male). The results show a significant difference between the regions of the CS and SNpc for LAMP-1 and LAMP-2A in DHM (**a**) and Sal B (**b**) treated groups and the saline-control group. The levels of LAMP-1 and LAMP-2A were significantly increased, while the levels of α-syn-GFP (the product of the transgene) were significantly decreased by DHM and Sal B treatments. However, the levels of mouse endogenous α-syn were not significantly different among the groups. Significant differences are indicated as DHM (**c**) and Sal B (**d**) treatments in the CS and SNpc compared with the saline treated group. All data shown are representative of at least three independent experiments (mean ± SD, *p<0.05, **p<0.01)

To further explore alterations of α-syn as well as LAMP-1 and LAMP-2A levels in BAC-α-syn-GFP transgenic mice, we performed SDS-PAGE and Western blotting (Fig. 7). We observed that levels of LAMP-1 and LAMP-2A were significantly up-regulated in DHM (Fig. 7a, c) and Sal B treated mice (Fig. 7b, d) in the CS and SNpc compared to the saline treated group, whereas levels of α-syn-GFP were significantly down-regulated following DHM and Sal B treatments. In addition, no changes were observed in the levels of the endogenous mouse α-syn.

### DHM and Sal B treatment lead to decreased astrogliosis and microgliosis *in vivo*

**Fig. 8:**
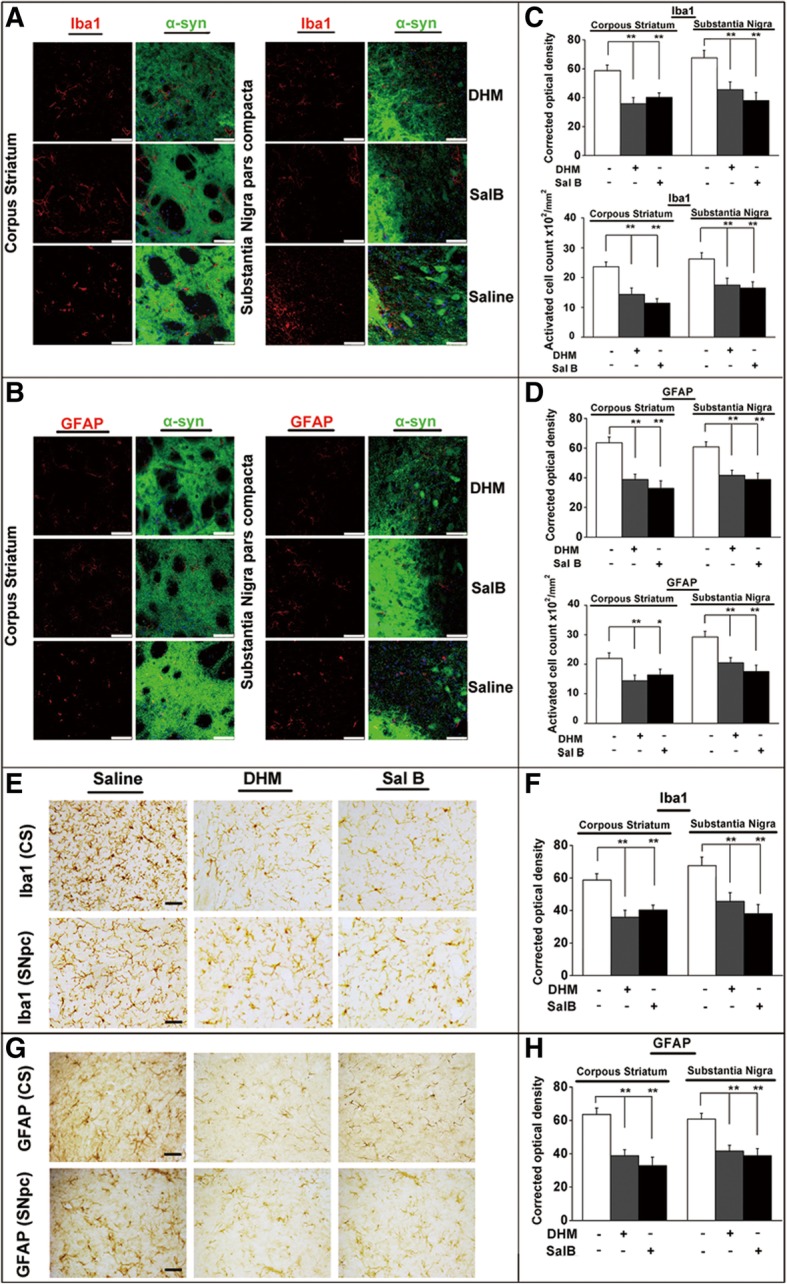
Activation of microglia and astrocytes in transgenic mice after DHM and Sal B treatments. Alterations in cell numbers of microglia are concomitant to that of astrocytes (**a** and **b**). Representative images of the microglial marker Iba1 (**a**) and the astroglial marker GFAP (**b**) in the CS and SNpc of BAC-α-syn-GFP transgenic mice treated with DHM/Sal B or saline control (8 animals/group, male). Quantification of activated cell numbers and density of the microglial marker Iba1 (**c**) and the astroglial marker GFAP (**d**) in the CS and SNpc of BAC-α-syn-GFP transgenic mice are displayed with treatments of DHM/Sal B or saline control. IHC assessment of astrogliosis paralleled by microgliosis in transgenic mice after DHM and Sal B treatments (**e**-**h**). Representative images of Iba1+ microglia (**e**) and GFAP+ astroglia (**g**) in the CS and SNpc of BAC-α-syn-GFP transgenic mice treated with DHM and Sal B or saline. Effects of DHM and Sal B treatments in the CS and SNpc of BAC-α-syn-GFP transgenic mice compared to saline treatment are represented by quantification of optical density of the microglial marker Iba1 (**f**) and the astroglial marker GFAP (**h**) in each individual group. All values are mean + S.E.M, differences are significant at *p<0.05, **p < 0.01. Scare bar = 50 μm

To investigate the effects of DHM and Sal B on inflammatory responses in the CS and SNpc, we examined the density and quantity of activated Iba1- and GFAP- positive cells in the substantia nigra and the striatum after DHM and Sal B treatments in BAC-α-syn-GFP transgenic mice. We observed a reduction in astrogliosis and microgliosis in both the CS and SNpc (Fig. 8a, b) by quantifying the number of activated astroglial cells and microglial cells as well as the astroglial and microglial density of IF staining results in BAC-α-syn-GFP transgenic mice (Fig. 8c, d). Quantification of the Iba1 (Fig. 8e, f) and GFAP (Fig. 8g, h) signal intensity of IHC staining also revealed a deactivation of astroglia and microglia by DHM and Sal B treatments in the CS as well as in the SNpc. These data suggest that DHM and Sal B treatments have anti-neuroinflammatory effects in BAC-α-syn-GFP transgenic mice.

## Discussion

In this study, we have shown that DHM and Sal B induce the degradation of α-syn aggregates and we attribute this to the observed activation of the CMA pathway both *in vitro* and *in vivo.* Firstly, low doses of DHM and Sal B could reduce the expression level of α-syn aggregates by up-regulating the CMA pathway in the SynT-aggregation cell model. We then confirmed that DHM and Sal B up-regulate LAMP-1, an important marker for the structure and function of lysosomal membranes and LAMP-2A, a key marker of CMA, in α-syn transgenic mice and decrease astrogliosis and microgliosis. This data indicates that treatment with DHM or Sal B upregulates the CMA pathway, which is known to play a key role in degrading abnormal a-syn aggregates.

Autophagy has been considered an essential mechanism in neurodegenerative diseases such as PD and Alzheimer’s disease [39–41]. Increasing evidence suggests that aggregated and misfolded α-syn drives the pathology of PD. Although it has been reported that Sal B can inhibit Aβ aggregation in cultured cells [24, 42], no evidence exists to indicate whether DHM or SalB have a regulatory effect on α-syn aggregation. Here, we observed decreased α-syn expression in cell models after DHM and Sal B treatments, as well as decreased levels of the α-syn protein in α-syn transgenic mice. Therefore, it is possible that the degradation pathway of aggregated α-syn may be directly targeted by DHM and Sal B. From a structural chemistry point of view, several studies have provided evidence that compounds which have three adjacent dihydroxy groups (e.g. DHM) or vicinal dihydroxy groups (e.g. Sal B) are effective inhibitors of α-syn oligomerization and fibrilization [43–45]. Thus, the special structures of DHM and Sal B may have a direct inhibitory effect on the aggregation of α-syn.

Lysosomes are the primary compartment for the degradation of intracellular proteins via autophagy [46]. The existence of abnormal intracellular α-syn-positive aggregates in PD indicates that the degradation capability of lysosomes may be impaired [3, 47]. CMA exerts a protective function by selectively targeting damaged or misfolded proteins for lysosomal degradation. Dysfunction of CMA in PD is characterized by reduced expression of the membrane receptor of CMA, lysosomal-associated membrane protein (LAMP) [4, 48, 49]. Several studies using different cell culture models of synucleinopathies have shown that the CMA pathway participates in α-syn degradation and its alteration may support α-syn mediated neurodegeneration [3, 7, 50]. Most of the previous studies report increased accumulation of α-syn by inhibiting CMA pathway, or reduced α-syn levels by activating CMA pathway [39, 51]. LAMP-2A plays an important role in the CMA pathway of α-syn degradation and an increased expression of LAMP-2A can activate the CMA pathway [4, 13, 52]. Here, we observed a reduction of α-syn aggregation by DHM and Sal B *in vitro*. The aggregation cell model is characterized by ThS-positive α-syn aggregates, because the dye can specifically bind to amyloid-like structures to indicate the formation of large inclusions [36, 53]. Smaller α-syn positive aggregates generated by untagged α-syn are also found in cell models, but are not positive for ThS. Thus, our data suggest that DHM and Sal B not only enhance α-syn degradation by the CMA pathway, but also modulate α-syn aggregation. Rapamycin has been widely used to inhibit the mTOR pathway and thereby induce autophagy [54]. Chloroquine blocks lysosomal function by raising lysosomal pH, thereby inhibiting lysosomal function [55]. Here, blocking autophagy with CQ resulted in SynT aggregation and increased toxicity. However, ALP modulation by rapamycin did not increase the toxicity of SynT and α-syn aggregation was reduced. We can see a clear effect of DHM and Sal B treatment on LAMP-1 and LAMP-2A levels, and a similar effect on aggregation induced by rapamycin. Notably, we observed that both LAMP-1 and LAMP-2A clearly co-localized with α-syn in transgenic mice after administration of DHM and Sal B, which is in agreement with previous findings [48, 56]. Recent studies showed that DHM and Sal B can enhance the level of autophagy by regulating the mTOR pathway [31, 57]. Sal B can stabilize the lysosome membrane by increasing the LAMP-1 protein level by reducing lysosomal enzyme translocation to the cytosol [58]. Levels of LAMP-1 can be increased through the regulation of the nuclear localization of Transcription factor EB (TFEB) via the mTOR signaling pathway [59]. LAMP-2A and the mTOR complex were highly relevant *in vivo* [60]. Thus, we reasoned that DHM and Sal B may enhance the degradation of α-syn by up-regulating the level of CMA and enhancing the expression of LAMP-1 and LAMP-2A via the mTOR signaling pathway.

As previously reported, DHM exerts a more rapid effect in association with enhancement of brain-derived neurotrophic factor expression and inhibition of neuroinflammation [61]. Administration of Sal B significantly decreased microglial activation in the central nervous system [62], promoted autophagy and induced the clearance of inflammasome, resulting in neuroprotective actions [63]. In our study, we demonstrated that both DHM and Sal B treatments effectively inhibited astroglia- and microglia-mediated neuroinflammation. It appears that DHM and Sal B can penetrate the blood-brain barrier and display multiple pharmacological activities, including oxidation resistance, anti-tumor properties and neuroprotection [23, 64], indicating potential for clinical application [65]. Both DHM and Sal B displayed a protective role towards dopaminergic neurons by exerting neuroprotective effects [66, 67].

## Conclusions

Through small molecule screening, we have identified two small molecules, DHM and Sal B that can inhibit α-synuclein aggregation in cell-free conditions. In α-synuclein overexpressing cell and animal models, we have demonstrated that both DHM and Sal B can inhibit α-synuclein accumulation and aggregation in cells and mouse brains. Decreasing α-synuclein aggregates concomitantly activates CMA pathways by increasing expression of LAMP-2A and macroautophagy by increasing LC3-II and LAMP-1, and is accompanied by the inhibition of microglial activation and neuroinflammation. Our results show that DHM and Sal B are effective in modulating α-synuclein accumulation and aggregate formation and augment CMA and macroautophagy. Furthermore, many chemotherapeutic agents have been reported to induce CMA activation, suggesting that autophagic protein degradation could be a potential approach to prevent and treat synucleinopathies. Our study strongly suggests that these two compounds may represent a detoxification and anti-inflammatory mechanism which could be targeted for clinical interventions of PD caused by abnormal accumulation and aggregation of α-syn.

## Additional files

Additional file 1: SynT-aggregation model and WT α-syn control. A 93 aa long C-terminal tag fused to α-syn (SynT) (Ai) and synphilin-1 were transiently co-transfected to H4 cells and resulted in larger intracellular α-syn inclusions. Immunostaining for α-syn revealed large inclusions that were ThS-positive (Bi, Bii). Smaller aggregates were ThS-negative (Bi, Bii). Transfection with untagged human WT α-syn (WT α-Syn) (Aii) did not result in larger α-syn immunopositive inclusions (Ci, Cii). (TIF 2251 kb)

Additional file 2: Expression of LC3-II and LAMP-2A in α-syn transfected H4 cells in response to treatments with DHM, Sal B and 3-MA. A control group with wildtype α-syn plasmid transfection was performed as ‘WT α-syn’. SynT and synphilin 1 co-transfection was performed as the experimental group with DHM, Sal B, and 3-MA treatments. (A) The levels of SynT, LC3-II and LAMP-1/-2A were measured by Western blots. Quantitative analyses of α-syn (SynT), LC3-II and LAMP-1/-2A normalized to β-actin (B-E) (n = 5). (B) SynT levels were increased even after treatment with 3-MA in DHM or Sal B treated groups. (C) 3-MA led to a decrease in LC3-II levels in SynT transfected H4 cells and the level of LC3-II was recovered after treating with DHM or Sal B. (D-E) LAMP-1 and LAMP-2A expression levels were increased in DHM or Sal B treated cells as compared to the 3-MA-treated SynT cells. ^*^ shows the comparison between SynT/P and DHM/3-MA and Sal B/3-MA, while ^#^ shows the comparison between 3-MA and DHM/3-MA and Sal B/3-MA. All data shown are representative of at least three independent experiments (mean ± SD, *p<0.01, ^#^p<0.01). (TIF 1633 kb)

Additional file 3: Open field tests showing the locomotor function of 6 and 9 month old mice after DHM and Sal B treatments. Ambulatory movement for (A) 6 month old and (B) 9 month old WT (wt), or homozygous (tg/tg) mice (8 animals/group, male), recorded for 15 min each. Two groups of homozygous (tg/tg) mice (8 animals/group, male) received intraperitoneal administrations of 5 mg/kg DHM/Sal B. (TIF 1594 kb)

Additional file 4: α-Syn-GFP co-localizes with CMA markers in transgenic mice. α-Syn was expressed with GFP (green) and subjected to immunocytochemistry for LAMP-1 and LAMP-2A (red) followed by confocal microscopic analyses. In the presence of overexpressed α-syn-GFP, a greater quantity of LAMP-1 and LAMP-2A co-localize with α-syn in the DHM and Sal B treated group compared to the saline treated group in the SNpc of BAC-α-syn-GFP transgenic mice (8 animals/group, male). White arrows point to lysosomes where α-syn-GFP and LAMP-1/-2A are co-localized. Scale bar = 50 μm. (TIF 5592 kb)

## Data Availability

All data generated or analyzed during this study are included in this published article [and its supplementary information files].

## Abbreviations

3-methyladenine: 3-MA
ALP: Autophagy-lysosomal pathway
BAC-α-syn-GFP: Bacterial Artificial Chromosome α-synuclein-green fluorescent protein
CMA: Chaperone-mediated autophagy
CQ: Chloroquine
CS: Corpus striatum
DHM: Dihydromyricetin
GFAP: Glial fibrillary acidic protein
HSC/Hsc70: Heat shock (70kDa) protein
Iba1/AIF1: Allograft inflammatory factor 1
IF: Immunofluorescence
IHC: Immunohistochemical
LAMP-1: Lysosomal-associated membrane protein 1
LAMP-2A: Lysosomal-associated membrane protein 2, isoform A
LDH: Lactate dehydrogenase
mTOR: Mammalian target of rapamycin
MTT: 3-(4,5-dimethyl-2-thiazolyl)-2,5-diphenyl-2-H-tetrazolium bromide
PD: Parkinson’s disease
PVDF: Polyvinylidene difluoride
RT: Room temperature
Sal B: Salvianolic acid B
SDS-PAGE: Sodium dodecyl sulfate polyacrylamide gel electrophoresis
SNpc: Substantia nigra pars compacta
SynT: C-terminal modified α-synuclein
ThS: Thioflavin S
ThT: Thioflavin T
UPS: Ubiquitin-proteasome system
WT α-syn: Wild-type α-synuclein
α-syn/α-Syn: Alpha-synuclein
