# Supplementary figures and images for: Dihydromyricetin and Salvianolic acid B inhibit alpha-synuclein aggregation and enhance chaperone-mediated autophagy
Source: Transl Neurodegener. 2019 Jun 15;8:18. doi: 10.1186/s40035-019-0159-7 (PMC6570948; doi:10.1186/s40035-019-0159-7)

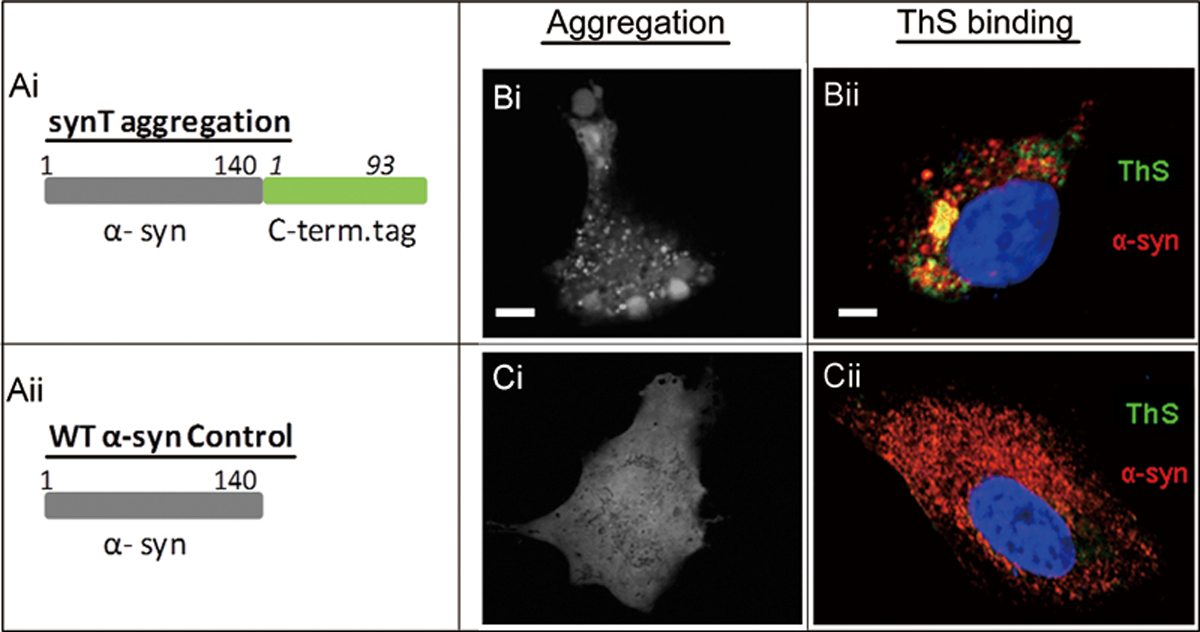

Supplement: Supplementary file 1 — SynT-aggregation model and WT α-syn control. A 93 aa long C-terminal tag fused to α-syn (SynT) (Ai) and synphilin-1 were transiently co-transfected to H4 cells and resulted in larger intracellular α-syn inclusions. Immunostaining for α-syn revealed large inclusions that were ThS-positive (Bi, Bii). Smaller aggregates were ThS-negative (Bi, Bii). Transfection with untagged human WT α-syn (WT α-Syn) (Aii) did not result in larger α-syn immunopositive inclusions (Ci, Cii). (TIF 2251 kb) [file 40035_2019_159_MOESM1_ESM.tif]

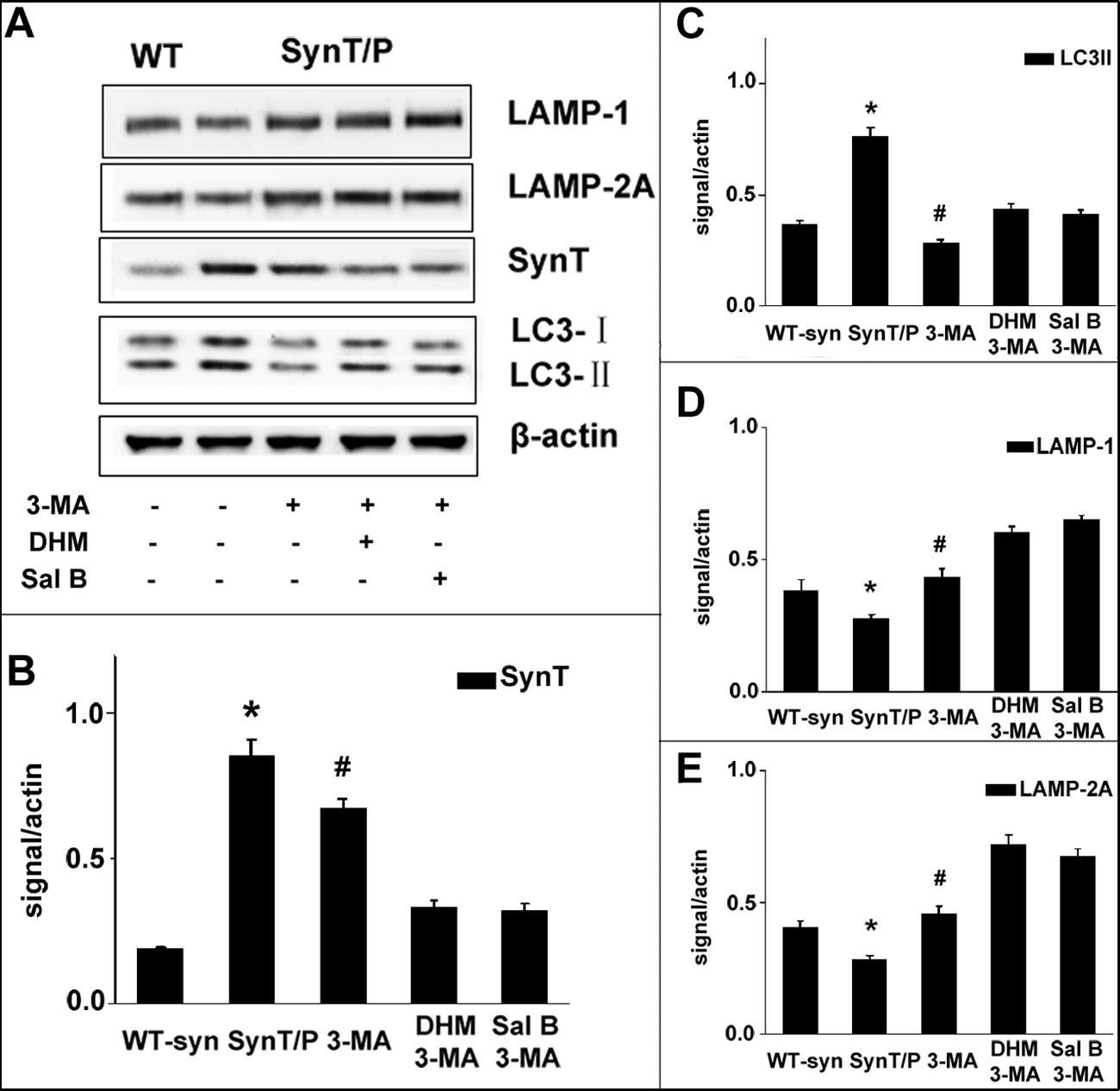

Supplement: Supplementary file 2 — Expression of LC3-II and LAMP-2A in α-syn transfected H4 cells in response to treatments with DHM, Sal B and 3-MA. A control group with wildtype α-syn plasmid transfection was performed as ‘WT α-syn’. SynT and synphilin 1 co-transfection was performed as the experimental group with DHM, Sal B, and 3-MA treatments. (A) The levels of SynT, LC3-II and LAMP-1/-2A were measured by Western blots. Quantitative analyses of α-syn (SynT), LC3-II and LAMP-1/-2A normalized to β-actin (B-E) (n = 5). (B) SynT levels were increased even after treatment with 3-MA in DHM or Sal B treated groups. (C) 3-MA led to a decrease in LC3-II levels in SynT transfected H4 cells and the level of LC3-II was recovered after treating with DHM or Sal B. (D-E) LAMP-1 and LAMP-2A expression levels were increased in DHM or Sal B treated cells as compared to the 3-MA-treated SynT cells. * shows the comparison between SynT/P and DHM/3-MA and Sal B/3-MA, while # shows the comparison between 3-MA and DHM/3-MA and Sal B/3-MA. All data shown are representative of at least three independent experiments (mean ± SD, *p<0.01, #p<0.01). (TIF 1633 kb) [file 40035_2019_159_MOESM2_ESM.tif]

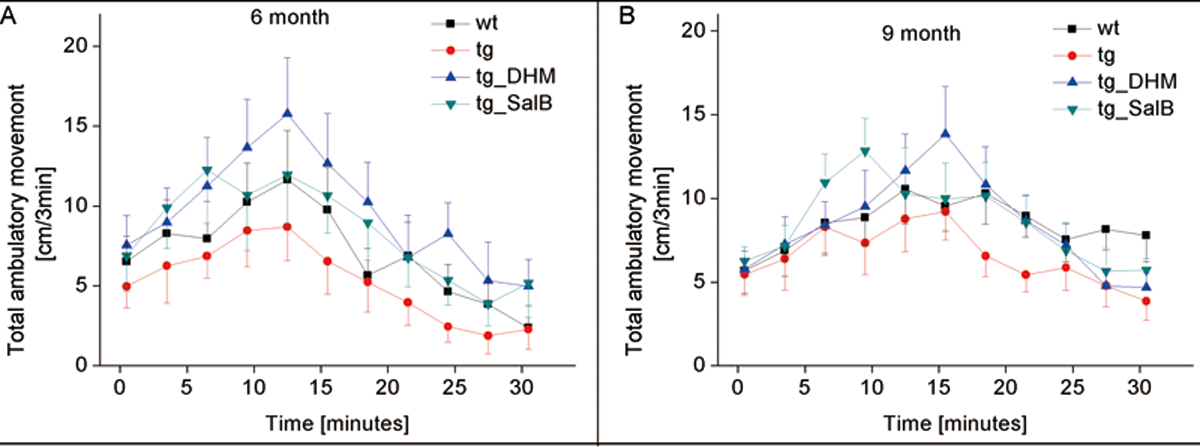

Supplement: Supplementary file 3 — Open field tests showing the locomotor function of 6 and 9 month old mice after DHM and Sal B treatments. Ambulatory movement for (A) 6 month old and (B) 9 month old WT (wt), or homozygous (tg/tg) mice (8 animals/group, male), recorded for 15 min each. Two groups of homozygous (tg/tg) mice (8 animals/group, male) received intraperitoneal administrations of 5 mg/kg DHM/Sal B. (TIF 1594 kb) [file 40035_2019_159_MOESM3_ESM.tif]

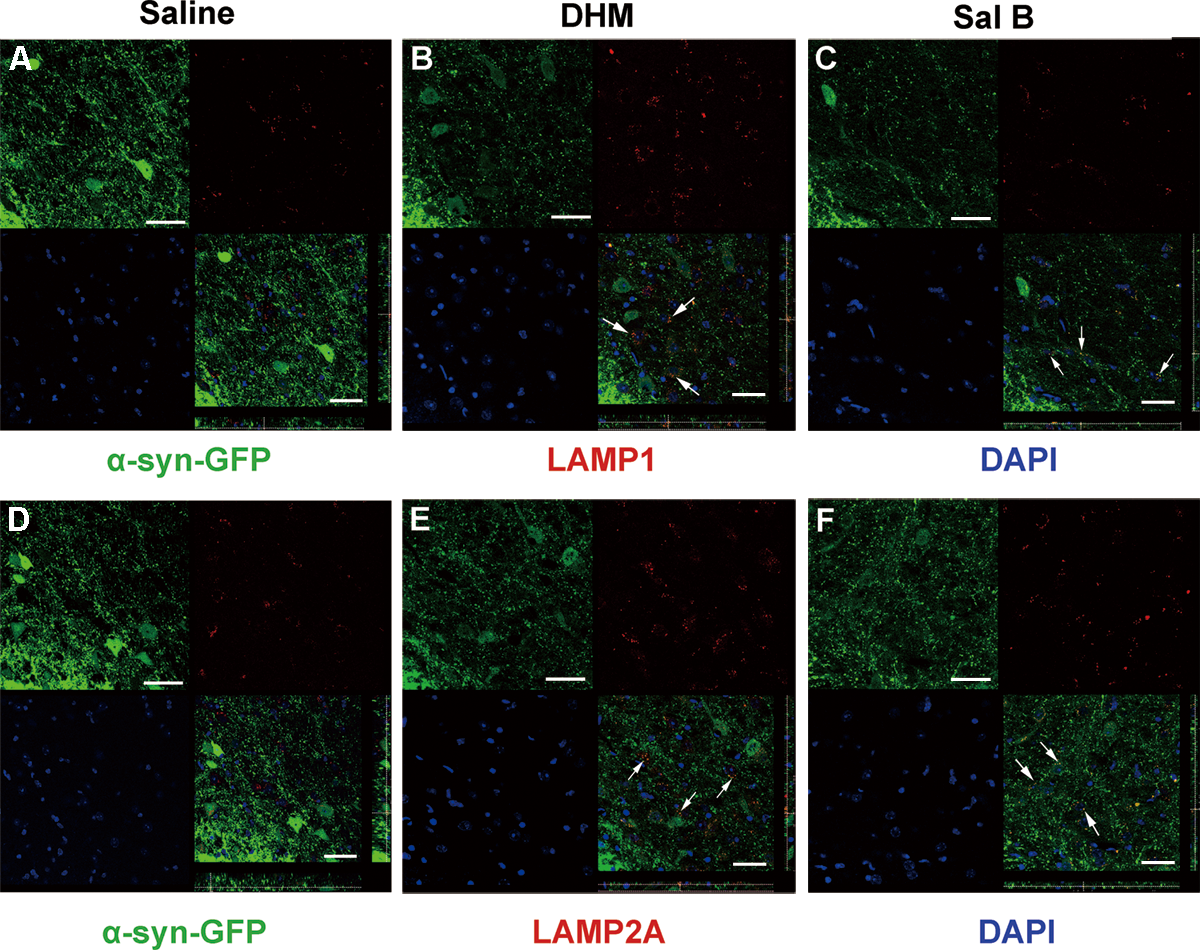

Supplement: Supplementary file 4 — α-Syn-GFP co-localizes with CMA markers in transgenic mice. α-Syn was expressed with GFP (green) and subjected to immunocytochemistry for LAMP-1 and LAMP-2A (red) followed by confocal microscopic analyses. In the presence of overexpressed α-syn-GFP, a greater quantity of LAMP-1 and LAMP-2A co-localize with α-syn in the DHM and Sal B treated group compared to the saline treated group in the SNpc of BAC-α-syn-GFP transgenic mice (8 animals/group, male). White arrows point to lysosomes where α-syn-GFP and LAMP-1/-2A are co-localized. Scale bar = 50 μm. (TIF 5592 kb) [file 40035_2019_159_MOESM4_ESM.tif]
